# mTOR controls endoplasmic reticulum–Golgi apparatus trafficking of VSVg in specific cell types

**DOI:** 10.1186/s11658-021-00262-z

**Published:** 2021-05-18

**Authors:** Alicja Koscielny, Ewa Liszewska, Katarzyna Machnicka, Michalina Wezyk, Katarzyna Kotulska, Jacek Jaworski

**Affiliations:** 1 International Institute of Molecular and Cell Biology, 4 Ks. Trojdena St., 04-421 Warsaw, Poland; 2 Laboratory of Neurogenetics, Department of Neurodegenerative Disorders, Mossakowski Medical Research Centre of the Polish Academy of Sciences, 5 Pawinskiego St., 02-106 Warsaw, Poland; 3 Department of Neurology and Epileptology, The Children’s Memorial Health Institute, Aleja Dzieci Polskich 20, 04-730 Warsaw, Poland

**Keywords:** MTOR, Endoplasmic reticulum, Golgi apparatus, VSVg, Tuberous sclerosis complex, Retention using selective hooks

## Abstract

**Background:**

Mammalian/mechanistic target of rapamycin (mTOR) complexes are essential for cell proliferation, growth, differentiation, and survival. mTORC1 hyperactivation occurs in the tuberous sclerosis complex (TSC). mTORC1 localizes to the surface of lysosomes, where Rheb activates it. However, mTOR was also found on the endoplasmic reticulum (ER) and Golgi apparatus (GA). Recent studies showed that the same inputs regulate ER-to-GA cargo transport and mTORC1 (e.g., the level of amino acids or energy status of the cell). Nonetheless, it remains unknown whether mTOR contributes to the regulation of cargo passage through the secretory pathway.

**Methods:**

The retention using selective hooks (RUSH) approach was used to image movement of model cargo (VSVg) between the ER and GA in various cell lines in which mTOR complexes were inhibited. We also investigated VSVg trafficking in TSC patient fibroblasts.

**Results:**

We found that mTOR inhibition led to the overall enhancement of VSVg transport through the secretory pathway in PC12 cells and primary human fibroblasts. Also, in TSC1-deficient cells, VSVg transport was enhanced.

**Conclusions:**

Altogether, these data indicate the involvement of mTOR in the regulation of ER-to-GA cargo transport and suggest that impairments in exocytosis may be an additional cellular process that is disturbed in TSC.

**Supplementary Information:**

The online version contains supplementary material available at 10.1186/s11658-021-00262-z.

## Introduction

Mammalian/mechanistic target of rapamycin (mTOR) is a protein kinase that controls cellular metabolism responding to trophic factors, nutrients, and cellular energy status [1–3]. These mTOR functions are primarily executed by mTOR complex 1 (mTORC1), which controls transcription, translation, protein stability, and subsequently cell growth and differentiation [1, 4]. mTORC2 regulates actin dynamics and the activity of AGC kinases (including A, G, and C kinase families) other than ribosomal S6 kinase beta-1 (S6K1) [5, 6]. mTORC2 regulates cell proliferation, migration, and survival. A distinctive molecular feature of mTORC1 is the presence in the complex of the Raptor protein. In mTORC2, the characteristic and specific partner of mTOR is Rictor. Raptor and Rictor define the substrate specificity of mTORC1 and mTORC2, respectively, and thus their cellular functions. However, mTOR complexes have been shown to have additional cellular functions, some of which may overlap. Among the most intensively studied aspects of mTORC biology are their intracellular localization and impact on mTORC activity and organelle functions. The best understood function in this regard is the importance of mTORC1 localization to the lysosomal surface, which acts as a coincidence detector. Following amino acid starvation, amino acid reappearance activates Ras-regulated GTP-binding proteins (Rags), which recruit mTORC1 to the lysosomal surface where it interacts with its upstream activator Ras homolog enriched in brain (Rheb) [7]. Active mTORC1 can affect lysosomal function in two ways. Once amino acid starvation ends, acting on UV radiation resistance-associated gene protein, mTORC1 ensures the restoration of lysosomes from autolysosomes [8]. On the other hand, mTOR at the transcriptional level downregulates lysosomal biogenesis, inhibiting movement of transcription factor EB (TFEB) to the nucleus [9]. Disruption of mTOR signaling, particularly mTORC1, is observed in many disorders commonly known as mTORopathies, e.g. tuberous sclerosis complex (TSC) [4, 10]. In TSC, loss of *TSC1* or *TSC2* leads to mTORC1 activity upregulation, substantial increases in cell soma size and several ultrastructural changes. Yet, endoplasmic reticulum (ER) and Golgi apparatus (GA) and the communication between these compartments are not thoroughly studied in TSC. Nevertheless, cells lacking TSC1–TSC2 complex are more vulnerable to ER stress and may display some disturbances in exocytosis [11–13].

Evidence suggests that both mTORCs are also located on the surface of other organelles apart from lysosomes. In mammalian cells, mTOR localizes to the GA and ER, which is the preceding compartment in the secretory pathway [14–16]. Such localization appears to be vital for the activation of S6K1, a canonical mTORC1 effector [16]. Recent studies in HEK293E cells showed that another small GTPase (i.e., Rab1A), rather than Rags, recruits mTORC1 to the GA, where it meets Rheb [17]. It is unknown whether mTORC1 activity affects ER-Golgi function either locally or by regulating the biosynthesis of their building components at the transcriptional or translational level. mTORC2 was also shown to be present on the ER, where it regulates Akt activity [14], which is an important kinase for ER cargo exit [18].

The ER and GA are essential parts of the secretory pathway, which directs newly synthesized proteins either toward the cell surface or other organelles. In mammalian cells, the secretory pathway, in addition to the ER and GA, consists of the ER-Golgi intermediate compartment (ERGIC), the trans-Golgi network (TGN), and secretory vesicles. ERGIC is an intermediate structure between the ER and GA, whereas TGN and secretory vesicles are post-GA organelles. The traffic of cargo between secretory pathway compartments occurs in both directions and is tightly controlled by protein kinases and small GTPases [19]. Akt is a kinase that acts on the coat protein complex II (COPII) and is needed for ER cargo exit [20, 21], thus regulating the efficiency of these processes [18]. Importantly, mTORC2 activates Akt, but its impact on the dynamics of cargo transport from the ER remains unknown. Akt phosphorylates Sec24, an essential part of the formation of so-called COPII vesicles that transport cargo from the ER toward the GA (or ERGIC; i.e., anterograde transport). Akt-phosphorylated Sec24 binds Sec23 more effectively, which should result in more efficient anterograde trafficking [18]. Another kinase that regulates the efficiency of transport within the secretory pathway is 5ʹ-adenosine monophosphate (AMP)-activated protein kinase (AMPK), which links low cellular energy levels to the effectiveness of secretion [22]. The primary mechanism of AMPK-dependent regulation involves the phosphorylation of Golgi-specific brefeldin A-resistance guanine nucleotide exchange factor 1 (GBF1), an Arf1 GAP that regulates COPI, a complex that is critical for retrograde transport from the GA to ER. However, low energy levels and AMPK are well-known negative regulators of mTORC1 [23, 24]. Another negative regulator of mTORC1, amino acid starvation, also affects ER-to-GA transport [25, 26]. Considering these observations, one issue is whether mTORC1 regulates this process. To address this issue, we used the retention using selective hooks (RUSH) method to image ER-to-GA transport and investigated the effects of mTOR inhibition and tuberous sclerosis complex 1 (TSC1) deficiency, which is known to induce mTORC1 hyperactivation [10], on VSVg, a prototypical cargo trafficking in the ER-to-GA pathway.

## Material and methods

### DNA constructs and lentiviral particle production

The plasmids pLKO.1-TRC cloning vector (Addgene plasmid no. 10878) and pLKO.1-TRC control vector (Addgene plasmid no. 10879) were a gift from Dr. D. Root [27]. pMD2.G and psPAX2 plasmids (Addgene plasmids # # 12259, 12260) were a gift from Didier Trono. shRNA against human TSC1 mRNA (GenBank no. NM_000368.4) was described previously [28] and inserted into the pLKO.1-TRC cloning vector. Str-Ii_VSVGwt-SBP-EGFP (Addgene plasmid no. 65300) was a gift from Dr. F. Perez [29]. Lentiviral vectors were produced and purified as described previously [30].

### Antibodies and drugs

The following primary antibodies were used: mouse anti-GM130 (catalog no. 610822; BD Biosciences, San Jose, CA, USA), mouse anti-tubulin (catalog no. T5168; Sigma-Aldrich, St. Louis, MO, USA), rabbit anti-hamartin/TSC1 (catalog no. 4906; Cell Signaling Technology, Danvers, MA, USA), rabbit anti-phospho-S6 ribosomal protein (Ser235/236; catalog no. 4858; Cell Signaling Technology), and rabbit anti-phospho-Akt (Ser473; catalog no. 4060; Cell Signaling Technology). Alexa Fluor 647-conjugated secondary antibody (anti-mouse) was purchased from Thermo Fisher Scientific (Waltham, MA, USA). Anti-mouse/anti-rabbit IRDye 680RD- and IRDye 800CW-conjugated antibodies were obtained from LI-COR Biosciences (Lincoln, NE, USA). INK128 and D-biotin were obtained from Selleckchem (Houston, TX, USA) or Sigma-Aldrich, respectively.

### Cell line culture, transfection, and transduction

Cell lines were obtained from Sigma Aldrich (HeLa cells) and the American Type Culture Collection (ATCC; Manassas, VA, USA; MCF7 and PC12 cells), respectively. Human fibroblasts were obtained from the shagreen skin patch of a TSC patient who carried a mutation of *TSC1* (*TSC1* c.1729G > T p.E577*). Control fibroblasts were obtained from a sex-matched healthy donor. The study was approved by the local Ethics Committee at the Children's Memorial Health Institute, Warsaw, Poland. Both patients and controls gave their written informed consent before the skin biopsy was performed. The skin biopsies were cut into 10–15 pieces, transferred to culture dishes, and cultured for 3 weeks. The culture medium was changed weekly. Cell outgrowth was then trypsinized and passaged to obtain a homogeneous culture of fibroblast cells. The presence of a mutation in the cultured patients’ fibroblasts was further confirmed by sequencing. HeLa cells, MCF7 cells, and fibroblasts were grown in Dulbecco’s modified Eagle’s medium (DMEM) that contained 10% fetal bovine serum (FBS) and 1% penicillin–streptomycin (Sigma-Aldrich). PC12 cells were grown in RPMI-1640 medium (Sigma-Aldrich) that contained 10% horse serum (Thermo Fisher Scientific), 5% FBS, and 1% penicillin–streptomycin (Sigma-Aldrich) until transfection, after which the medium was replaced with DMEM containing 10% FBS and 1% penicillin–streptomycin (Sigma-Aldrich) to avoid biotin presence. The cells were cultured at 37 °C in a 5% CO_2_ atmosphere on 13 or 18 mm glass coverslips for immunofluorescence or live imaging experiments, respectively. Before cell seeding, the coverslips were covered for 20 min with 0.2% aqueous gelatin solution (Sigma-Aldrich; for HeLa cells, MCF7 cells, and fibroblasts) or with 0.05 mg/ml poly-D-lysine (Sigma-Aldrich; PC12 cells). The cells were transfected using polyethylenimine (catalog no. 23966; Polysciences, Warrington, PA, USA; HeLa cells and MCF7 cells) or Lipofectamine2000 (Thermo Fisher Scientific; PC12 cells) or electroporated using a CUY21 device (Nepagene, Chiba, Japan; fibroblasts) with Str-Ii_VSVGwt_SBP_EGFP according to the manufacturer’s protocols. For the knockdown experiments, lentiviral vectors were produced as described above, and added to the fibroblasts for 12 h (250 μl/well in a six-well plate). Next, the culture medium was replaced with fresh medium, and cells were returned to the cell culture incubator.

### Western blot

Western blot and quantitative analysis with the Odyssey Infrared Imaging System (LI-COR Biosciences) were performed as described previously [30]. Images of all uncropped Western blot membranes from the whole study can be found in Additional file 14.

### Immunofluorescence and imaging of fixed cells

HeLa cells, MCF7 cells, PC12 cells, and fibroblasts were fixed and immunofluorescently stained as described previously [30]. Images of immunofluorescently labeled cells were acquired using a Zeiss NLO 710 confocal microscope at 1024 × 1024 pixel resolution. Oil objectives (63 × [1.5 × zoom] or 100 ×) were used for imaging HeLa cells, MCF7 cells, and fibroblasts or PC12 cells, respectively. Z-stacks of the images were averaged twice per line and then converted to single images with a maximum intensity projection function. The settings were kept constant for all of the scans.

### RUSH assay and image analysis

Twenty-four hours after transfection, cells that were grown on glass coverslips were moved to a low-profile open bath chamber (RC-41LP; Warner Instruments, Hamden, CT, USA) containing DMEM (in the case of all analyzed cell types). For the experiments in which we inhibited mTOR activity, the cells were treated with 300 nM INK128 for 30 min before live imaging. At time 0 min, the cells were treated with 40 mM D-biotin. Time-lapse imaging was performed at 37 °C in a 5% CO_2_ atmosphere using a spinning-disc microscope (Andor Revolutions XD, Belfast, UK) and a thermostat-controlled chamber. Oil objectives (63 × or 100 ×) were used for imaging HeLa cells, MCF7 cells, and fibroblasts or PC12 cells, respectively. Images were acquired once per minute for 1 h (HeLa cells and MCF7 cells), every 2 min for 1 h (PC12 cells), and every 2 min for 1.5 h (fibroblasts). Z-stacks were generated at 1004 × 1002 pixel resolution for HeLa cells, MCF7 cells, and PC12 cells and 502 × 501 pixel resolution for fibroblasts, and next converted to 2D images with a maximum intensity projection method. For all of the scans, the microscope settings were kept constant. ImageJ software (National Institutes of Health, Bethesda, MD, USA) was used for image analysis. The image alignment was done with the StackReg plug-in for ImageJ [31]. Next, three different regions of interest of the same size that contained the GA were selected, and the total fluorescence intensity was measured for each time point using the Time Series Analyzer v3 plug-in (Balaji J., Department of Neurobiology, University of California, Los Angeles, CA, USA) for ImageJ and normalized to the maximum value [1]. The position of the GA was defined based on cell images that were obtained at later time points.

### Statistical analysis

The analyzed numbers of cells (*n*) and culture batches (*N*) and types of statistical analyses used are described in the figure legends.

## Results

### Endoplasmic reticulum-GA transport of VSVg is mTOR-independent in HeLa and MCF7 cells

Previous studies showed that mTOR is present on the surface of secretory pathway membranes, and such localization might be needed at least partially for its activity. It remains unknown whether mTOR influences secretory trafficking. We first tested the effect of INK128, an adenosine triphosphate-competitive inhibitor of mTOR that influences the activity of both mTORC1 and mTORC2, on cargo trafficking through the ER and GA. We used the RUSH method [29, 32] to visualize the trafficking of model cargo (vesicular stomatitis virus G protein [VSVg]) in living HeLa cells. In this method, the addition of free biotin, which disrupts the interaction between streptavidin-tagged ER anchor (Ii protein), and VSVg that is fused to streptavidin binding peptide and green fluorescent protein (GFP), triggers trafficking of the latter in the secretory pathway, which can be visualized by spinning-disc microscopy [29, 32]. We chose to study HeLa cells because the RUSH system was optimized with this cell line, and these cells are often used for studies that focus on mTOR.

**Fig. 1:**
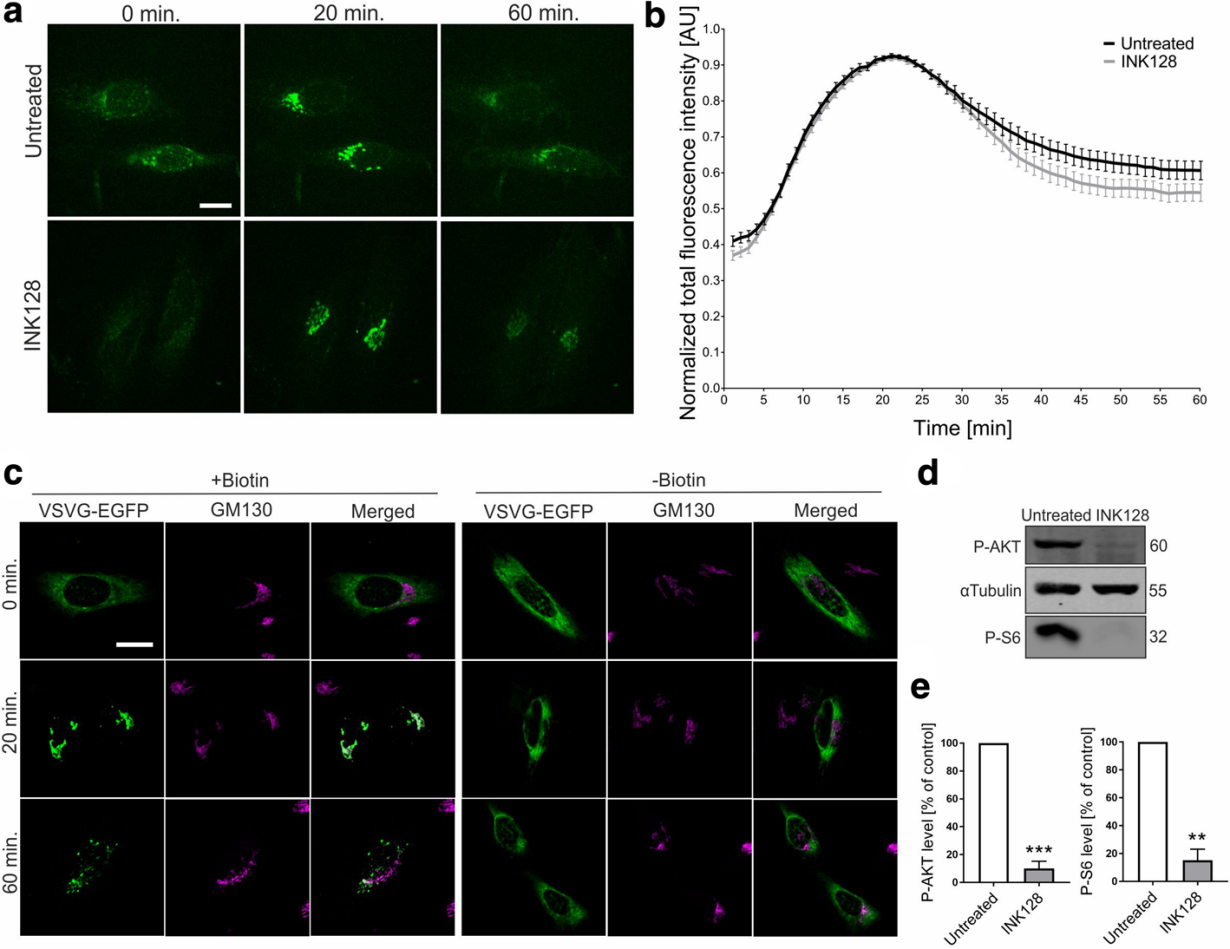
Inhibition of mTOR does not influence ER-to-GA VSVg trafficking in early-passage HeLa cells.** a** Representative time-lapse confocal images of living HeLa cells that were transfected with Str-li_VSVGwt-SBP-EGFP and treated with INK128 (300 nM, 30 min) or untreated (control). Trafficking of VSVg-EGFP was visualized using the RUSH assay for 60 min after the addition of biotin (time 0). Scale bar = 20 µm. **b** Quantitative analysis of experiments performed as in (**a**). The graph depicts VSVg-EGFP fluorescence intensity in the Golgi apparatus region at each time point, normalized to the maximum value. The data are expressed as the mean for all of the analyzed cells. Error bars indicate SEM. *N* = 4 independent experiments. Number of cells per variant (*n*): Untreated (35), INK128 (45). **c** Representative confocal images of HeLa cells that were transfected with Str-li_VSVGwt-SBP-EGFP (green) and immunofluorescently stained for the *cis*-Golgi marker GM130 (magenta). Scale bar = 20 µm. **d** Western blot analysis of phospho-AKT (P-AKT) and phospho-S6 (P-S6) levels in protein lysates from control HeLa cells or cells after INK128 treatment (300 nM, 30 min). **e** Quantification of Western blot analysis of P-AKT and P-S6, normalized to tubulin, in protein lysates that were obtained from HeLa cells that were treated as in (**d**). **p < 0.01, ***p < 0.001 (one-sample *t*-test). *N* = 4 independent experiments

HeLa cells were transfected with a plasmid that encoded all elements of the RUSH system (Str-Ii_VSVGwt_SBP_EGFP) for 24 h. Next, biotin (40 µM) was added to the culture media and VSVg-enhanced GFP (EGFP) was imaged for 1 h with 1 min intervals with spinning-disc microscopy (Fig. 1; Additional file 2: Movie S1). At the 0 min time point, no GFP fluorescence was detected in the GA, but ~ 10 min after the addition of biotin, VSVg-EGFP localized to this compartment, reaching maximum fluorescence at 20 min (Fig. 1a, b). When biotin was omitted from the culture media, the change in GFP signal localization was not observed (Additional file 1: Fig. S1). To confirm the localization of VSVg to the GA, we performed an analogous experiment but fixed cells at 0, 20, and 60 min after the addition of biotin and stained them with the GA marker GM130. At the 0 min time point, the VSVg-EGFP signal did not colocalize with GM130 immunofluorescence. At the 20 min time point, the two signals strongly overlapped (Fig. 1c). At the 60 min time point, the overlapping signals were weaker, suggesting that VSVg-EGFP exited the GA. As expected, when biotin was not added, no overlapping signals were observed at any of the time points (Fig. 1c). When INK128 (300 nM) was added to the cells 30 min before the addition of biotin, no difference with the control variant was observed (Fig. 1a, b; Additional file 2: Movie S1, Additional file 3: Movie S2), although INK128 treatment alone decreased levels of phosphorylated ribosomal protein S6 (serines 235 and 236, P-S6) and Akt (threonine 473, P-Akt; Fig. 1d, e; see also Additional file 14 for images of uncropped Western blot membranes), which are widely used indicators of mTORC1 and mTORC2 activity, respectively.

To confirm our observations in a different cell line, we performed experiments that were analogous to HeLa cells using the tumor cell line MCF7. In these cells, VSVg-EGFP fluorescence peaked in the GA slightly faster than in HeLa cells (15 vs. 20 min), but pretreatment with INK128 did not affect VSVg transport in the secretory pathway (Additional file 1: Fig. S2; Additional file 4: Movie S3, Additional file 5: Movie S4). Based on these results, we concluded that mTOR activity in HeLa and MCF7 cells is not needed for VSVg transport through the ER and GA.

### mTOR inhibition affects VSVg transport through the ER-GA in PC12 cells and human fibroblasts

**Fig. 2:**
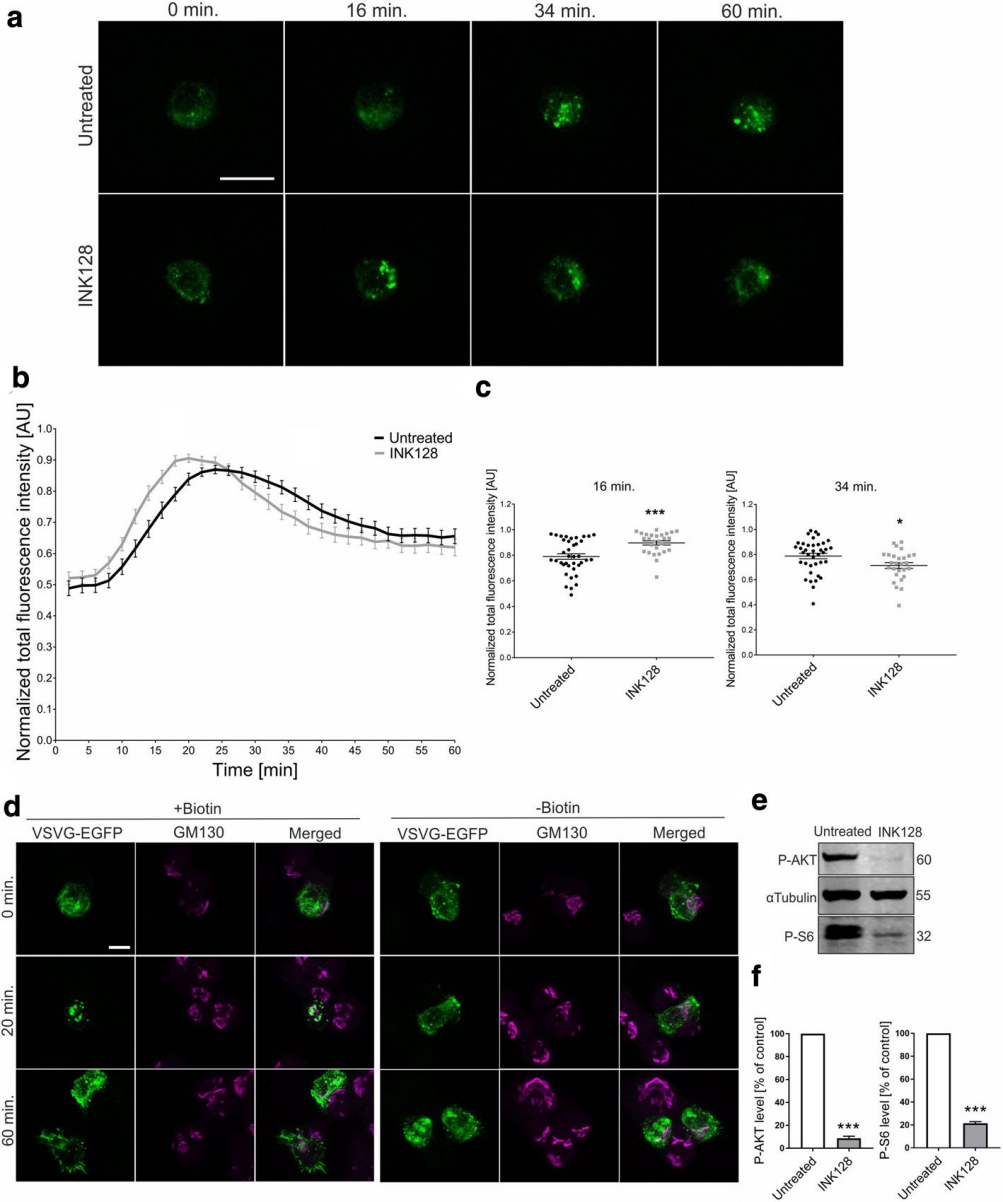
Inhibition of mTOR affects ER-to-GA VSVg trafficking in PC12 cells.** a** Representative time-lapse confocal images of living PC12 cells that were transfected with Str-li_VSVGwt-SBP-EGFP and treated with INK128 (300 nM, 30 min) or untreated (control). Trafficking of VSVg-EGFP was visualized using the RUSH assay for 60 min after the addition of biotin (time 0). Scale bar = 20 µm. **b** Quantitative analysis of the experiments performed as in (**a**). The graph depicts VSVg-EGFP fluorescence intensity in the Golgi apparatus region at each time point, normalized to the maximum value. The data are expressed as the mean for all of the analyzed cells. Error bars indicate SEM. *N* = 4 independent experiments. Number of cells per variant (*n*): Untreated (38), INK128 (27). **c** Comparison of VSVg-EGFP fluorescence intensity in the Golgi apparatus region at 16 min (left panel) and 34 min (right panel). The data are expressed as the mean for all of the analyzed cells. Error bars indicate SEM. *p < 0.05, ***p < 0.001 (Mann–Whitney test). The number of independent experiments and analyzed cells per variant are the same as in (**b**). **d** Representative confocal images of PC12 cells that were transfected with Str-li_VSVGwt-SBP-EGFP (green) and immunofluorescently stained for the *cis*-Golgi marker GM130 (magenta). Scale bar = 5 µm. **e** Western blot analysis of phospho-AKT (P-AKT) and phospho-S6 (P-S6) levels in protein lysates from control PC12 cells or cells after INK128 treatment (300 nM, 30 min). **f** Quantification of Western blot analysis of P-AKT and P-S6, normalized to tubulin, in protein lysates that were obtained from PC12 cells that were treated as in (**e**). ***p < 0.001 (one-sample *t*-test). *N* = 3 independent experiments

Although HeLa and MCF are cancer cells that are derived from different tissues, both cell lines are adenocarcinomas that have an epithelial origin. Thus, mTOR could still affect the secretory pathway in different cell types. To test this hypothesis, we used PC12 cells, which are derived from pheochromocytoma of the adrenal gland and are widely used to study secretion. PC12 cells were transfected with Str-Ii_VSVGwt_SBP_EGFP. After transfection, the medium was replaced with one lacking biotin, and the RUSH assay was performed 1 day after transfection (Fig. 2a; Additional file 6: Movie S5, Additional file 7: Movie S6). In control cells that were not treated with INK128, the maximal accumulation of VSVg-EGFP in the GA was reached after 24 min (Fig. 2a, b), which was additionally confirmed by the immunofluorescent staining of fixed cells (Fig. 2d). In the absence of biotin, no changes in VSVg distribution were observed throughout the duration of the experiment, similar to HeLa and MCF7 cells (Fig. 2d). However, unlike in HeLa and MCF7 cells, pretreatment with INK128 (30 min, 300 nM), which effectively diminished the phosphorylation of Akt and ribosomal protein S6 (Fig. 2e, f; see also Additional file 14 for images of uncropped Western blot membranes), resulted in faster accumulation of VSVg-EGFP fluorescence in the GA, reaching the most pronounced difference compared with control cells after 16 min (Fig. 2b, c). The point of maximum VSVg-EGFP fluorescence was reached earlier upon INK128 treatment (20 vs. 24 min; Fig. 2a, b; Additional file 6: Movie S5, Additional file 7: Movie S6). At the same time, the cargo left the GA earlier in INK128 cells compared with control cells (Fig. 2c, comparison of signal intensity in the GA at 34 min), suggesting a faster cargo transition through the ER-GA.

**Fig. 3:**
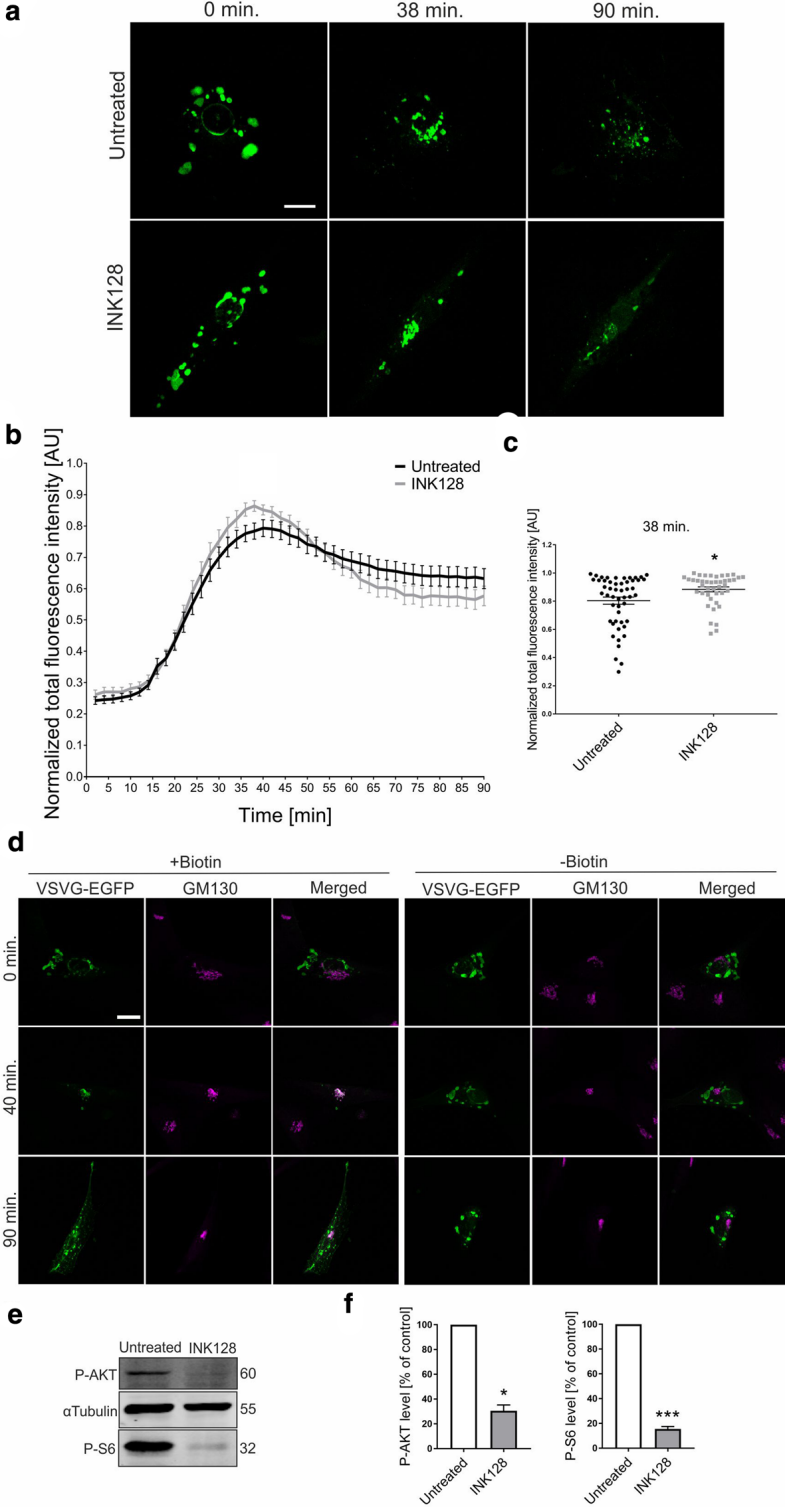
Inhibition of mTOR affects ER-to-GA VSVg trafficking in human fibroblasts. **a** Representative time-lapse confocal images of living fibroblasts that were electroporated with Str-li_VSVGwt-SBP-EGFP and treated with INK128 (300 nM, 30 min) or untreated (control). Trafficking of VSVg-EGFP was visualized using the RUSH assay for 90 min after the addition of biotin (time 0). Scale bar = 20 µm. **b** Quantitative analysis of the experiments performed as in (**a**). The graph presents VSVg-EGFP fluorescence intensity in the Golgi apparatus region at each time point, normalized to the maximum value. The data are expressed as the mean for all of the analyzed cells. Error bars indicate SEM. *N* = 3 independent experiments. Number of cells per variant (*n*): Untreated (51), INK128 (42). **c** Comparison of VSVg-EGFP fluorescence intensity in the Golgi apparatus region at 38 min. The data are expressed as the mean for all of the analyzed cells. Error bars indicate SEM. *p < 0.05 (Mann–Whitney test). The number of independent experiments and analyzed cells per variant are the same as in (**b**). **d** Representative confocal images of fibroblasts that were electroporated with Str-li_VSVGwt-SBP-EGFP (green) and immunofluorescently stained for the *cis*-Golgi marker GM130 (magenta). Scale bar = 20 µm. **e** Western blot analysis of phospho-AKT (P-AKT) and phospho-S6 (P-S6) levels in protein lysates from control fibroblasts or fibroblasts after INK128 treatment (300 nM, 30 min). **f** Quantification of Western blot analysis of P-AKT and P-S6, normalized to tubulin, in protein lysates that were obtained from fibroblasts treated as in **e**. *p < 0.05, ***p < 0.001 (one-sample *t*-test). Number of independent experiments (*N*): P-AKT (2), P-S6 (3)

Because mTOR appeared to influence secretory trafficking, depending on the cell line, we tested another cell type, primary human dermal fibroblasts. In control fibroblasts, the kinetics of VSVg transport through the ER-GA was much slower than in all other cell types tested. Maximum fluorescence was reached 38–40 min after the addition of biotin (Fig. 3a, b; Additional file 8: Movie S7, Additional file 9: Movie S8). We also noted that EGFP-VSVg fluorescence before biotin addition stained relatively large structures that could not be readily identified, some of which could, however, represent large ER packaging buds described recently by McCaughey et al. [33]. However, it should be noted that these structures did not affect the analysis of the presence of VSVg in GA because they disappeared with the administration of biotin before the cargo reached GA. In contrast to PC12 cells, treatment with INK128 did not substantially accelerate the accumulation of VSVg in the GA, but significantly more cargo accumulated at the peak of VSVg-EGFP fluorescence (38 min for INK128-treated cells; Fig. 3c). The difference between maximal VSVg-EGFP fluorescence and at 90 min (i.e., the end of imaging) was also more visible upon exposure to INK128, suggesting more efficient release, but the effect did not reach statistical significance (*p* = 0.062; Mann–Whitney test). We then confirmed that VSVg passed through the GA and INK128 affected mTORCs using immunofluorescence and Western blot, respectively (Fig. 3d–f; see also Additional file 14 for images of uncropped Western blot membranes). Altogether, these results suggest that mTOR inhibition results in the more rapid or efficient transition of VSVg through the ER-GA pathway in specific cell types, likely characterized by intensive exocytosis.

### Lack of TSC results in faster VSVg transport through the ER-GA

**Fig. 4:**
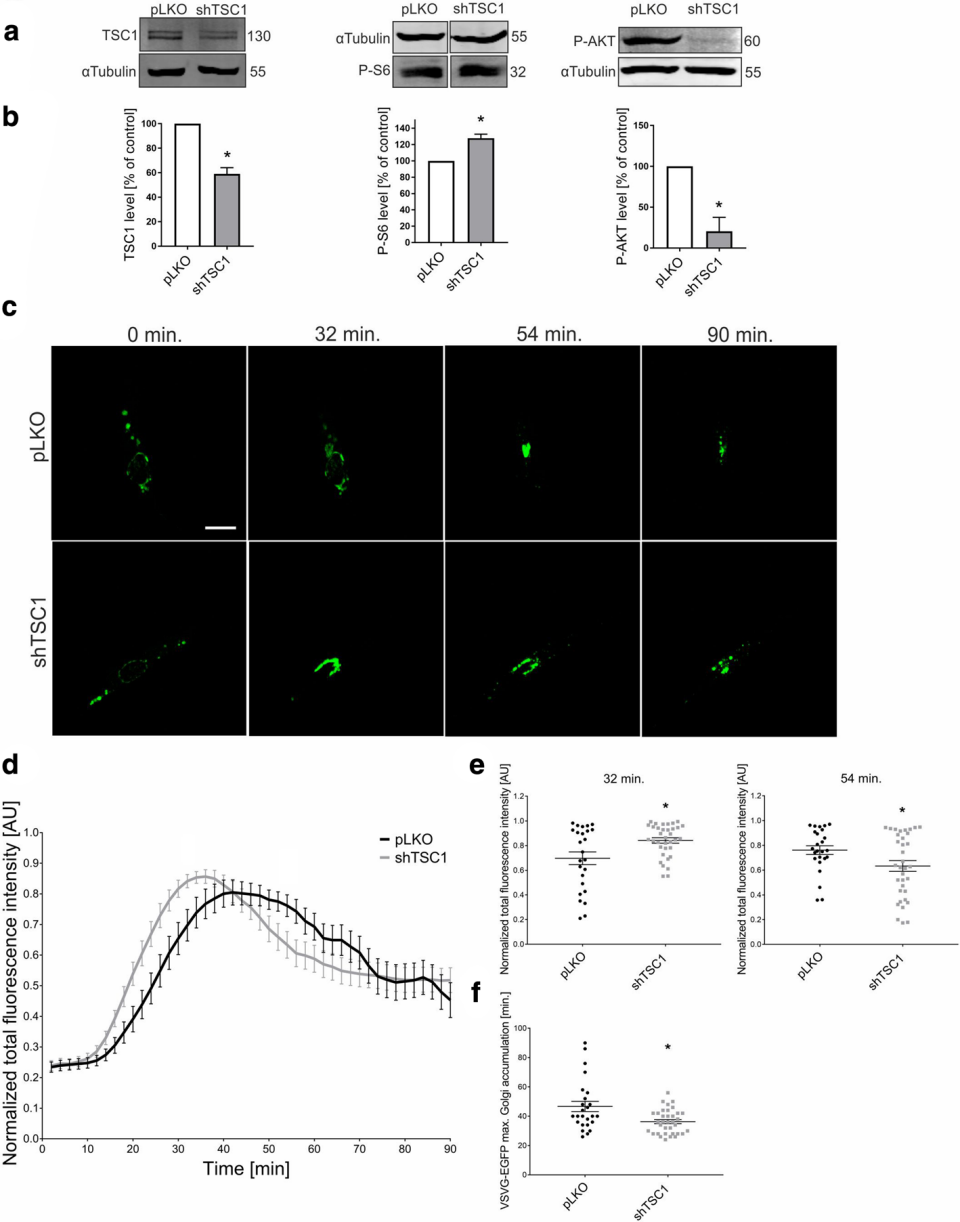
ER-to-GA VSVg trafficking is affected in human fibroblasts with TSC1 knockdown.** a** Western blot analysis of TSC1, phospho-S6 (P-S6), and phospho-AKT (P-AKT) levels in protein lysates from control pLKO and shTSC1 fibroblasts. **b** Quantification of Western blot analysis of hamartin, P-AKT, and P-S6, normalized to tubulin, in protein lysates that were obtained as in (**a**). *p < 0.05 (one-sample *t*-test). *N* = 3 independent experiments. **c** Representative time-lapse confocal images of living fibroblasts: control (pLKO) and with TSC1 knockdown (shTSC1). The fibroblasts were electroporated with Str-li_VSVGwt-SBP-EGFP. Trafficking of VSVg-EGFP was visualized using the RUSH assay for 90 min after the addition of biotin (time 0). Scale bar = 20 µm. **d** Quantitative analysis of the experiments performed as in (**c**). The graph presents VSVg-EGFP fluorescence intensity in the Golgi apparatus region at each time point, normalized to the maximum value. The data are expressed as the mean for all of the analyzed cells. Error bars indicate SEM. *N* = 3 independent experiments. Number of cells per variant (*n*): pLKO (25), shTSC1 (35). **e** Comparison of VSVg-EGFP fluorescence intensity in the Golgi apparatus region at 32 min (left panel) and 54 min (right panel). The data are expressed as the mean for all of the analyzed cells. Error bars indicate SEM. *p < 0.05 (Mann–Whitney test). The number of independent experiments and analyzed cells per variant are the same as in (**d**). **f** Comparison of VSVg-EGFP maximum accumulation times in the Golgi apparatus region. The data are expressed as the mean for all of the analyzed cells. Error bars indicate SEM. *p < 0.05 (Mann–Whitney test). The number of independent experiments and analyzed cells per variant are the same as in (**d**)

The findings above indicate that the inhibition of mTOR affects VSVg transition via the ER-GA. We then investigated whether the lack of TSC activity, which leads to the activation of mTORC1, has the opposite effect. We first transduced wildtype human dermal fibroblasts with a lentiviral vector (pLKO.1-TRC [27]) that encodes a previously validated short-hairpin RNA (shRNA) against *TSC1* mRNA [28]. Transduction resulted in the downregulation of TSC1 protein levels and upregulation of P-S6 levels compared with cells that were transduced with the control lentiviral vector pLKO.1-TRC-control [27] (Fig. 4a, b; see also Additional file 14 for images of uncropped Western Blot membranes). At the same time, P-Akt levels decreased compared with control cells (Fig. 4a, b; see also Additional file 14 for images of uncropped Western Blot membranes), which was consistent with previous findings in cells that lacked TSC1-TSC2 [34, 35]. Using these cells, we performed the RUSH assay (Fig. 4c; Additional file 10: Movie S9, Additional file 11: Movie S10). VSVg in cells that had lower TSC1 accumulated in the GA more rapidly (see fluorescence intensity comparison for 32 min), reaching maximum accumulation in the GA earlier (36 vs. 42 min), and left the GA significantly faster than in pLKO-transduced cells (Fig. 4e, f).

**Fig. 5:**
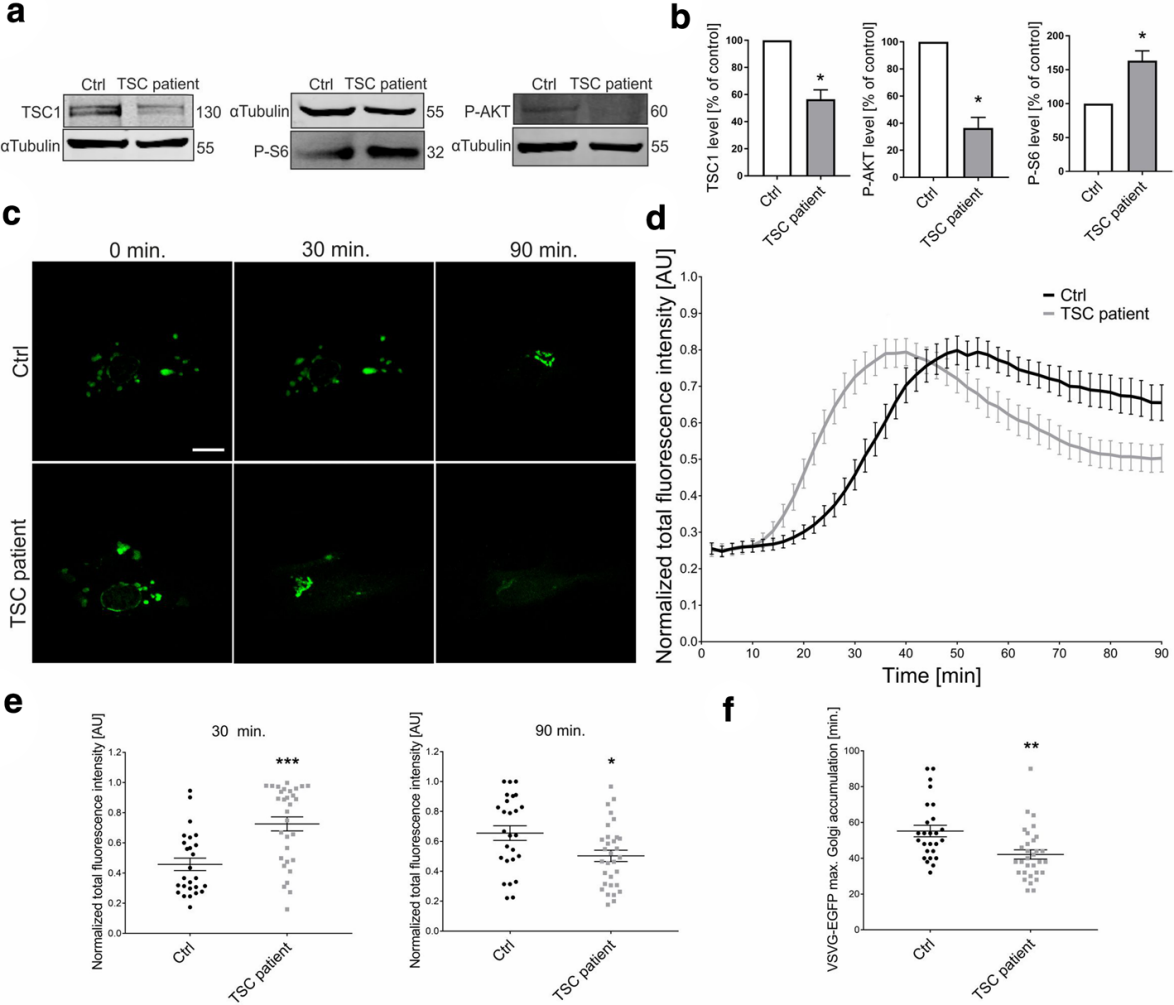
ER-to-GA VSVg trafficking is affected in fibroblasts from a TSC patient. **a** Western blot analysis of TSC1, phospho-S6 (P-S6), and phospho-AKT (P-AKT) levels in protein lysates from control fibroblasts (Ctrl) and fibroblasts from a TSC patient. **b** Quantification of Western blot analysis of P-AKT, P-S6, and TSC1, normalized to tubulin, in protein lysates that were obtained as in (**a**). *p < 0.05 (one-sample *t*-test). *N* = 3 independent experiments. **c** Representative time-lapse confocal images of living fibroblasts: control (Ctrl) and from a TSC patient. The fibroblasts were electroporated with Str-li_VSVGwt-SBP-EGFP. Trafficking of VSVg-EGFP was visualized using the RUSH assay for 90 min after the addition of biotin (time 0). Scale bar = 20 µm. **d** Quantitative analysis of the experiments performed as in **c**. The graph shows VSVG-EGFP fluorescence intensity in the Golgi apparatus region at each time point, normalized to the maximum value. The data are expressed as the mean for all of the analyzed cells. Error bars indicate SEM. *N* = 3 independent experiments. Number of cells per variant (*n*): Ctrl (26), TSC patient (31). **e** Comparison of VSVg-EGFP fluorescence intensity in the Golgi apparatus region at 30 min (left panel) and 90 min (right panel). The data are expressed as the mean for all of the analyzed cells. Error bars indicate SEM. *p < 0.05, ***p < 0.001 (Mann–Whitney test). The number of independent experiments and analyzed cells per variant are the same as in (**d**). **f** Comparison of VSVg-EGFP maximum accumulation times in the Golgi apparatus region. The data are expressed as the mean for all of the analyzed cells. Error bars indicate SEM. **p < 0.01 (Mann–Whitney test). The number of independent experiments and analyzed cells per variant are the same as in (**d**)

To further corroborate our observation that lower TSC activity in cells accelerates the transport of VSVg through the ER-GA, we performed the RUSH assay using dermal fibroblasts that carried a *TSC1* mutation (*TSC1* c.1729G > T p.E577*) that was derived from the shagreen patch of a TSC patient (Fig. 5; Additional file 12: Movie S11, Additional file 13: Movie S12). Fibroblasts that were derived from a healthy person served as a control. In cells with the *TSC1* mutation, the level of TSC1 was substantially lower, and levels of P-S6 and P-Akt changed accordingly (Fig. 5a, b; see also Additional file 14 for images of uncropped Western blot membranes). In TSC mutant cells, VSVg-EGFP accumulated in the GA more rapidly (Fig. 5c–e) and reached a maximum in the GA significantly faster than in control cells (~ 40 vs. 50 min; Fig. 5c, d, f). VSVg also exited from the GA earlier and more efficiently, which could be demonstrated by comparing the EGFP signal intensity at the experiment endpoint (90 min) between TSC patient and control cells (Fig. 5c–e). These data confirmed that the lack of TSC1 resulted in acceleration of the transition of VSVg through initial compartments of the secretory pathway.

## Discussion

Secretory trafficking was recently shown to be controlled by energy levels and adenosine monophosphate-activated protein kinase (AMPK) activity, which are both known for their impact on mTORC1 signaling. Thus, mTOR and the secretory pathway are controlled at least partially by the same factors. However, the contribution of mTOR to the control of cargo trafficking within this pathway has remained mostly unexplored. In the present study, using the RUSH method, we found that mTOR inhibition increased the effectiveness of the transport of VSVg, an archetypal secretory pathway cargo, into and out of the GA. Under basal culture conditions, this phenomenon occurred only in specific cell lines (i.e., PC12 cells and fibroblasts). Paradoxically, we also found that lower levels of TSC1, which resulted in the upregulation of mTORC1, also led to a similar phenotype in human fibroblasts. Before proceeding to further discussion, it should be clearly emphasized that our short report focuses mainly on the dynamics of VSVg transport to and from the GA using the RUSH technique. However, due to the limitations of this method, it does not allow us to conclude in which part of the GA, (i.e., cis-, medial-, or trans-) mTOR and TSC1-TSC2 complex exert their effects on cargo transport. Determining this will require further research using higher-resolution methods.

Previous studies have not specifically focused on the effects of mTOR inhibition on the secretory pathway. Nevertheless, evidence indicates that rapamycin, an mTORC1 inhibitor, decreases the secretion of interleukin-1α (IL-1α) in senescent cells [36]. The effects of rapamycin were attributed, however, to the inhibition of IL-1α synthesis. Thus, these studies do not contradict our results because differences in endogenous cargo expression should not affect the outcome of the RUSH assay. However, previous studies reported that the lack of TSC2 led to the arrest of various cargo proteins in the GA and post-GA compartments. Polycystin-1 levels at the plasma membrane (PM) decreased in rat kidney cells that lacked Tsc2 because of the accumulation of this protein in the GA [37]. The poor delivery of VSVg and caveolin-1 to the PM in rat embryonic fibroblasts that lacked Tsc2 was shown to result from disturbances of cytoskeleton dynamics and post-GA trafficking [12, 13]. The overexpression of constitutively active Rheb mimicked these effects, implying that mTORC1 hyperactivity adversely impacts post-GA trafficking [12]. In the present study, we did not analyze post-GA trafficking; therefore, the findings of the present study, Jones et al. [13], and Jiang and Yeung [12] should not be directly compared. One open question is why we observed an acceleration of VSVg trafficking into and out of the GA in human fibroblasts with low Tsc1 levels, whereas polycystin-1 was stopped at the GA in rat kidney cells that lacked Tsc2. Differences in experimental design may explain this discrepancy. For example, the present results suggest that cargo trafficking into and out of the GA is differentially regulated by mTOR in different cell types. Moreover, the control of different cargo transport through the secretory pathway is not identical. Thus, VSVg and polycystin-1 may differentially respond to the lack of TSC1 and TSC2 and further studies should focus on testing mTOR effects on a variety of available cargos for RUSH. Finally, although Tsc1 and Tsc2 act as one protein complex, the effects of Tsc1 and Tsc2 deficiency in the cell may differ. Phenotypes that are related to the loss of Tsc2 have been postulated to be more severe for several reasons, including stronger mTORC1 pathway activation or the engagement of TSC1 in protein complexes that are different from Tsc1- Tsc2 complex.

In the present study, mTOR inhibition by INK128 accelerates and/or enhances VSVg accumulation in the GA and release from this organelle. Short-term treatment of INK128 cells, in our experiments, had the definite advantage of helping to avoid secondary effects accompanying, for example, RNA interference or knockout experiments. At the same time, it allowed blockage of rapamycin-independent mTORC1 activity. Unfortunately, it also blocked mTORC2. Thus, we could not determine whether mTORC1, mTORC2, or both complexes are responsible for these phenomena. Therefore, further experiments involving genetic inactivation of mTORC1 or mTORC2 by deletion of Raptor or Rictor, respectively, will be necessary to determine which mTOR complex affects VSVg transport in selected cell types. As such, we only discuss potential options and arguments for and against below.

mTORC1 is primarily known for its role in translation and transcription. Thus, one could speculate that its inhibition affects the biogenesis of secretory pathway machinery. Although we cannot exclude such a possibility, the effects of INK128 were relatively rapid, suggesting that changes in biosynthesis of the ER and GA were unlikely the cause of the observed phenotype. However, in Tsc1-deficient cells, an increase in mTORC1 activity is a chronic state, and such a mechanism cannot be excluded. Another link between mTORC1 and secretory cargo trafficking between the ER and GA could involve autophagy regulation. For example, it was shown that autophagy induction, e.g., as a result of amino acid starvation, results in substantial GA fragmentation that could impact our results [38]. However, in our opinion, this phenomenon could not have been the primary cause of the differences we observed in VSVg transport in the secretory pathway. First, comparison images from individual films clearly showing the GA apparatus did not reveal significant differences between the compared experimental conditions within individual cell lines. Second, in our study, cells were treated with INK128 for only 30 min before biotin addition. For other ATP-competent mTOR inhibitors, e.g., Torin 1, no apparent changes in LC3-II levels were seen even up to 2 h after administration [39]. Of course, it should be noted that the referred study involved a different inhibitor and other cell type than our work. Finally, if Golgi structure change due to autophagy was the primary reason for the observed effect of INK128 on VSVg transport, it would be difficult to explain the lack of effect of INK128 in HeLa and MCF7 cells. Another link between ER to GA trafficking of VSVg, mTORC1 and autophagy could involve Unc-51-like autophagy-activating kinase (Ulk-1). Upon autophagy induction, Ulk-1 increases the effectiveness of COPII complexes that involve Sec16A and Sec24c and export the serotonin transporter SERT from the ER to the GA [26]. mTORC1 is a well-known negative regulator of Ulk-1, and mTORC1 inhibition could lead to more efficient Ulk-1-controlled COPII vesicle formation and release. However, the same study showed that amino acid starvation had no effect on VSVg trafficking [26]. Other studies showed that amino acid starvation or the Ulk1-dependent phosphorylation of Sec23A halted VSVg in the ER [25]. These two observations argue against a mechanism whereby mTORC1 inhibition accelerates VSVg transport via the initiation of autophagy. Thus, mTORC1 effectors other than Ulk1 would be responsible for the acceleration of VSVg transport from the ER to GA in INK128-treated cells. However, analyses of the mTOR phosphoproteome [40–43] have not revealed any apparent candidates, such as regulators of release and the docking of vesicles to and from the ER and GA. mTORC1 has been shown to regulate phosphorylation of the cytoplasmic linker protein Clip-170, a microtubule-binding protein. Clip-170 is important for the loading of p150^glued^ of the dynein–dynactin complex onto microtubule plus-ends and was postulated to be important for vesicle transport from the ER to the GA [44]. Subsequent studies, however, showed that Clip-170 is not needed for the functions of p150^glued^ in the stabilization of ER and GA structure and communication [45]. mTOR also phosphorylates other microtubule-binding proteins (e.g., microtubule-associated protein 1B [MAP1B]), the role of which in ER-to-GA transport was demonstrated in the case of the calcium ion-controlled transport of specific cargo [46]. It remains unknown whether MAB1B phosphorylation by mTORC1 affects MAP1B function in this regard.

Instead of, or in addition to, mTORC1, another possibility is that VSVg transport within the secretory pathway is regulated by mTORC2. Such a mechanism could explain why INK128 treatment and Tsc1 deficiency had similar effects on VSVg transport. However, it must be emphasized that, as in the case of cell lines with normal TSC1 levels, indicating whether the observed changes in VSVg transport depend on mTORC1 and mTORC2 (or on mTOR at all) requires further studies, preferably using appropriate molecular tools. Many studies have shown that, in addition to mTORC1 upregulation, the activity of some mTORC2 substrates in TSC1-TSC2 deficient cells (e.g., Akt) decreases (see also Fig. 5 and 6). As mentioned above, Akt is an essential regulator of COPII vesicle formation [18]. Recent studies in *C. elegans* showed that the knockdown of serum- and glucocorticoid-inducible kinase-1 (SGK1), another mTORC2 target, disturbed ER-GA transport [47]. However, if mTORC2 acts on ER-GA transport via these kinases, INK128 treatment, as well as Tsc1 deficiency, should result in decelerated secretory trafficking, which would be the opposite to the results reported herein. However, similar to mTORC1, mTORC2-dependent regulation of the cytoskeleton needs to be considered as one factor that influences the effectiveness of cargo transport through the secretory pathway. mTORC2 is a known regulator of actin dynamics [5, 6], which regulates cargo transport through the secretory pathway, both at the ER and GA [48].

An additional issue that needs to be considered is the different origins of the cell lines that were evaluated in the present study with regard to their secretory pathway sensitivity to mTOR inhibition. mTOR inhibition may not affect cells that are derived from adenocarcinomas of epithelial origin. Such an hypothesis may be supported by the fact that HeLa cells (early passage) and MCF7 cells are insensitive to INK128, whereas fibroblasts and PC12 cells respond to INK128. However, PC12 cells and fibroblasts are also intensively secreting cells, suggesting that mTOR may influence ER-to-GA VSVg transport only in actively secreting cells.

## Conclusions

Overall, the present study using the RUSH method showed that a prototypical secretory pathway cargo transport from ER to GA, depends on mTOR in specific cell types and lack of Tsc1, observed in TSC patients, may also impact this process, potentially contributing to TSC cellular phenotypes.

## Supplementary Information

**Additional file 1: Figure S1.** Without the addition of biotin, VSVg-EGFP resides in the endoplasmic reticulum of p8 HeLa cells. igure S2. Inhibition of mTOR does not influence secretory trafficking in early-passage MCF7 cells.

**Additional file 2: Movie S1.** Exemplary results of RUSH assay in control HeLa cells

**Additional file 3: Movie S2.** Exemplary results of RUSH assay in HeLa cells treated with INK128

**Additional file 4: Movie S3.** Exemplary results of RUSH assay in control MCF7 cells

**Additional file 5: Movie S4.** Exemplary results of RUSH assay in MCF7 cells treated with INK128

**Additional file 6: Movie S5.** Exemplary results of RUSH assay in control PC12 cells

**Additional file 7: Movie S6.** Exemplary results of RUSH assay in PC12 cells treated with INK128

**Additional file 8: Movie S7.** Exemplary results of RUSH assay in control primary fibroblasts

**Additional file 9: Movie S8.** Exemplary results of RUSH assay in primary fibroblasts treated with INK128

**Additional file 10: Movie S9.** Exemplary results of RUSH assay in control primary fibroblasts transfected with control pLKO vector

**Additional file 11: Movie S10.** Exemplary results of RUSH assay in primary fibroblasts transfected with pLKO vector encoding shTSC1

**Additional file 12: Movie S11.** Exemplary results of RUSH assay in control primary fibroblasts

**Additional file 13: Movie S12.** Movie S1. Exemplary results of RUSH assay in primary TSC patient fibroblasts

**Additional file 14:** Images of uncropped Western blot membranes

## Data Availability

The materials and datasets used and analyzed during the current study are available from the corresponding author on reasonable request.

## Abbreviations

AMPK: Adenosine monophosphate (AMP)-activated protein kinase
BSA: Bovine serum albumin
Clip-170: Cytoplasmic linker protein of 170 kD
COPII: Coat protein complex II
DMEM: Dulbecco’s modified Eagle’s medium
EGFP: Enhanced GFP
ER: Endoplasmic reticulum
ERGIC: ER-Golgi intermediate compartment
FBS: Fetal bovine serum
GA: Golgi apparatus
GBF1: Golgi-specific brefeldin A-resistance guanine nucleotide exchange factor 1
GFP: Green fluorescent protein
IL-1α: Interleukin-1α
MAP1B: Microtubule-associated protein 1B
mTOR: Mammalian/mechanistic target of rapamycin
mTORC1: MTOR complex 1
mTORC2: MTOR complex 2
P-Akt: Phosphorylated Akt (threonine 473)
PBS: Phosphate-buffered saline
PM: Plasma membrane
P-S6: Phosphorylated ribosomal protein S6 (serines 235 and 236,)
Rags: Ras-regulated GTP-binding proteins
Rheb: Ras homolog enriched in brain
RUSH: Retention using selective hooks
S6K1: Ribosomal S6 kinase beta-1
SGK1: Serum- and glucocorticoid-inducible kinase-1
shRNA: Short-hairpin RNA
TFEB: Transcription factor EB
TGN: Trans-Golgi network
TSC: Tuberous sclerosis complex
TSC1: Tuberous sclerosis complex 1 protein
TSC2: Tuberous sclerosis complex 2 protein
Ulk-1: Unc-51-like autophagy-activating kinase
VSVg: Vesicular stomatitis virus G protein [VSVg
