# Supplementary material for: mTOR controls endoplasmic reticulum–Golgi apparatus trafficking of VSVg in specific cell types
Source: Cell Mol Biol Lett. 2021 May 18;26:18. doi: 10.1186/s11658-021-00262-z (PMC8130434; doi:10.1186/s11658-021-00262-z)
Supplement: Supplementary file 5 — Additional file 5: Movie S4. Exemplary results of RUSH assay in MCF7 cells treated with INK128 [file 11658_2021_262_MOESM5_ESM.pdf]

mTOR controls endoplasmic reticulum-Golgi apparatus trafficking of VSVg in specific cell types

Alicja Kościelny, Ewa Liszewska, Katarzyna Machnicka, Michalina Wezyk, Katarzyna Kotulska & Jacek Jaworski

Images of uncropped Western blot membranes

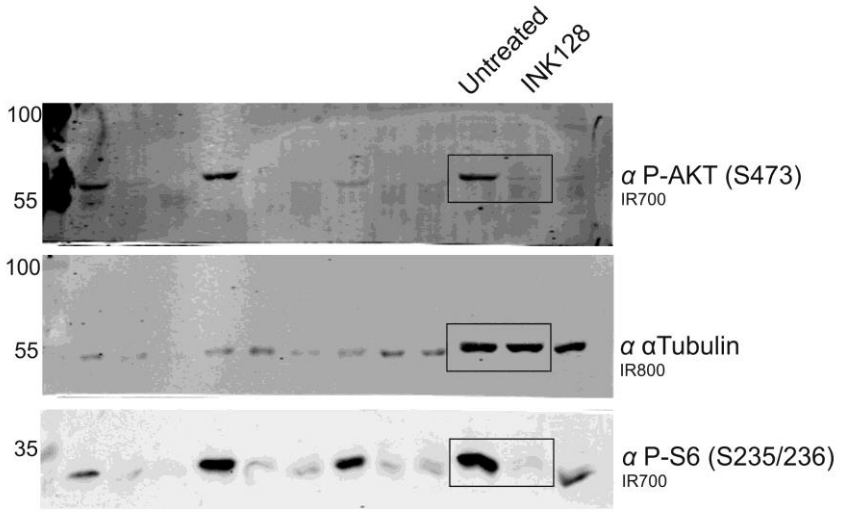

Fig. 1.

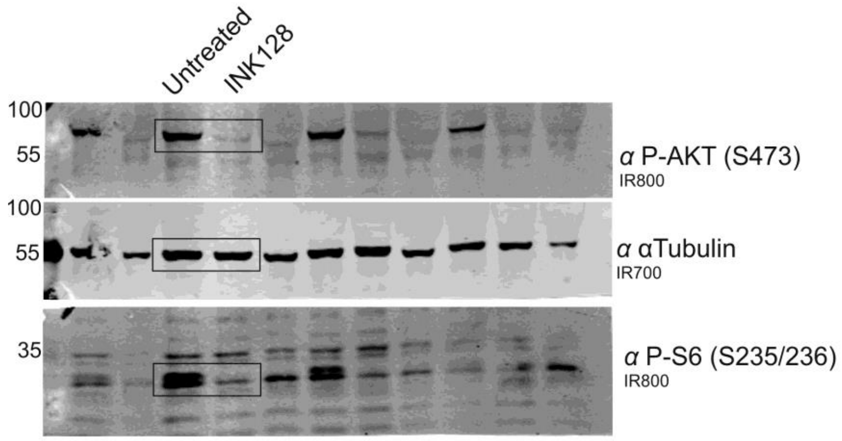

Fig. 2.

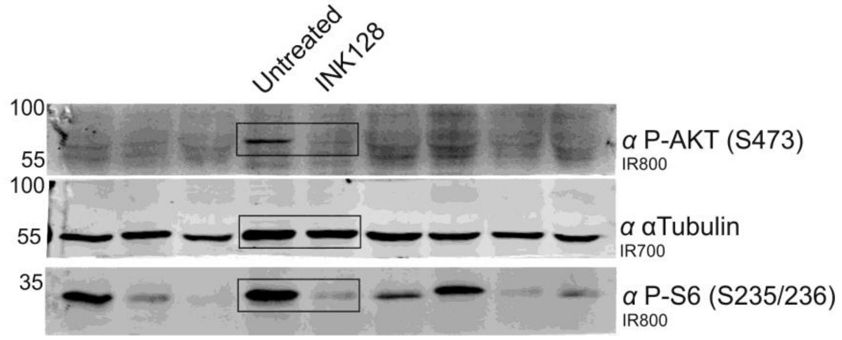
Untreated
INK128
100
55
α P-AKT (S473)
IR800
100
55
α αTubulin
IR700
35
α P-S6 (S235/236)
IR800

Fig. 3.

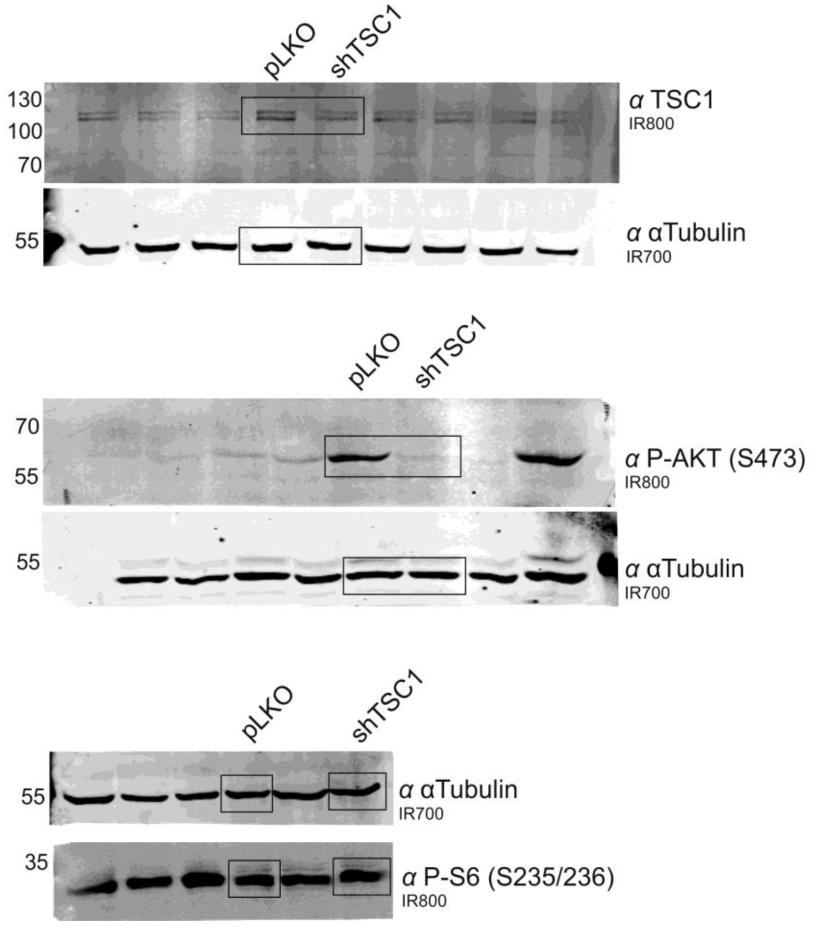
pLKO
shTSC1
130
100
70
α TSC1
IR800
55
α αTubulin
IR700
pLKO
shTSC1
70
55
α P-AKT (S473)
IR800
55
α αTubulin
IR700
pLKO
shTSC1
55
α αTubulin
IR700
35
α P-S6 (S235/236)
IR800

Fig. 4.

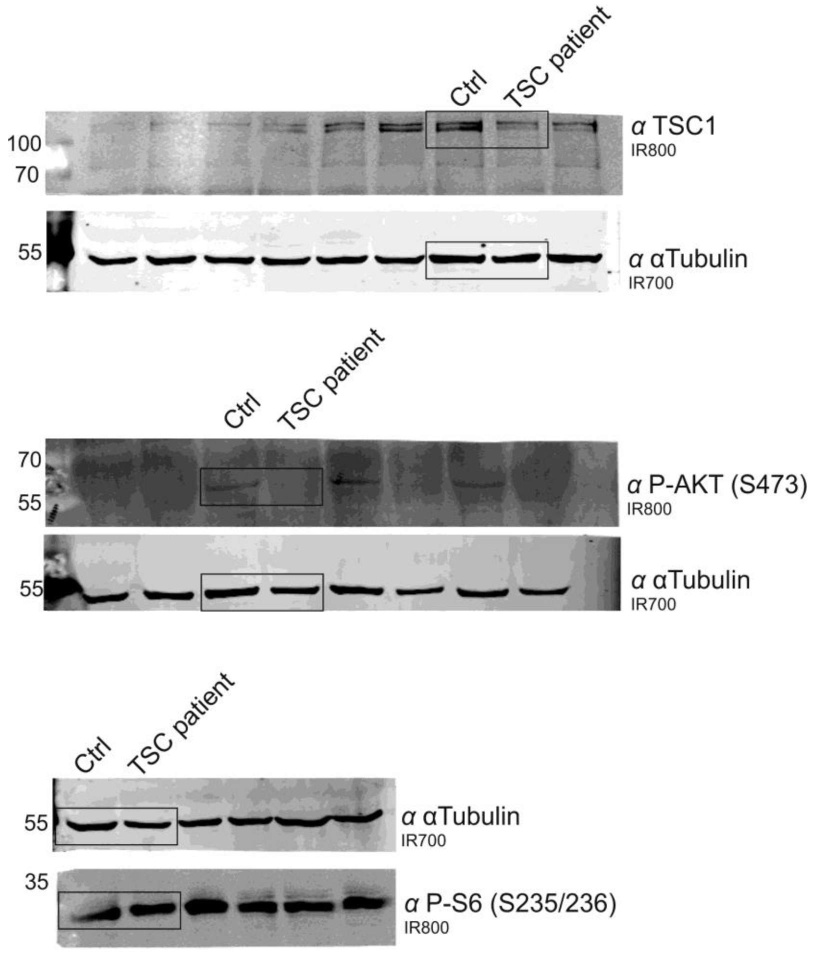

Fig. 5.

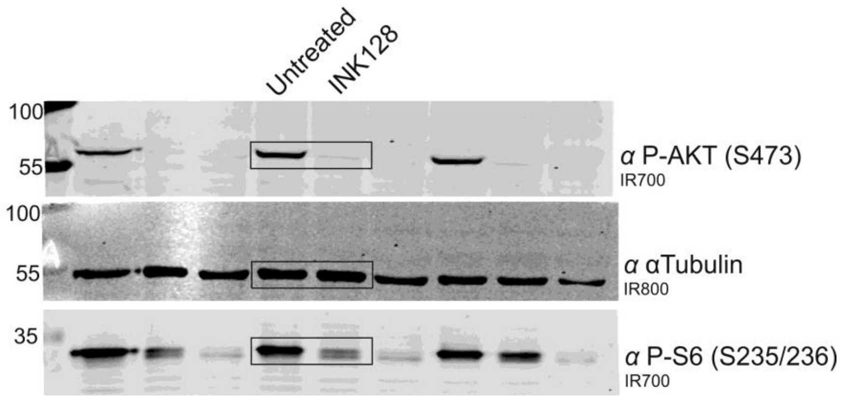

Additional Fig. S2
