# Supplementary material for: mTOR controls endoplasmic reticulum–Golgi apparatus trafficking of VSVg in specific cell types
Source: Cell Mol Biol Lett. 2021 May 18;26:18. doi: 10.1186/s11658-021-00262-z (PMC8130434; doi:10.1186/s11658-021-00262-z)
Supplement: Supplementary file 6 — Additional file 6: Movie S5. Exemplary results of RUSH assay in control PC12 cells [file 11658_2021_262_MOESM6_ESM.pdf]

Supplementary Information

mTOR controls endoplasmic reticulum-Golgi apparatus trafficking of VSVg in specific cell types

Alicja Kościelny, Ewa Liszewska, Katarzyna Machnicka, Michalina Wezyk, Katarzyna Kotulska & Jacek Jaworski

1. Supplementary Figures

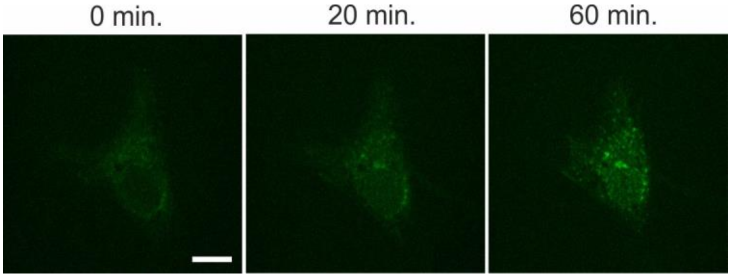

Additional Fig. S1. Without the addition of biotin, VSVg-EGFP resides in the endoplasmic reticulum of HeLa cells. Representative time-lapse confocal images of living HeLa cells that were transfected with Str-li_VSVGwt-SBP-EGFP and not treated with biotin. Secretory trafficking of the fluorescent reporter protein VSVg-EGFP was analyzed using the RUSH system for 60 min. Scale bar = 20 µm.

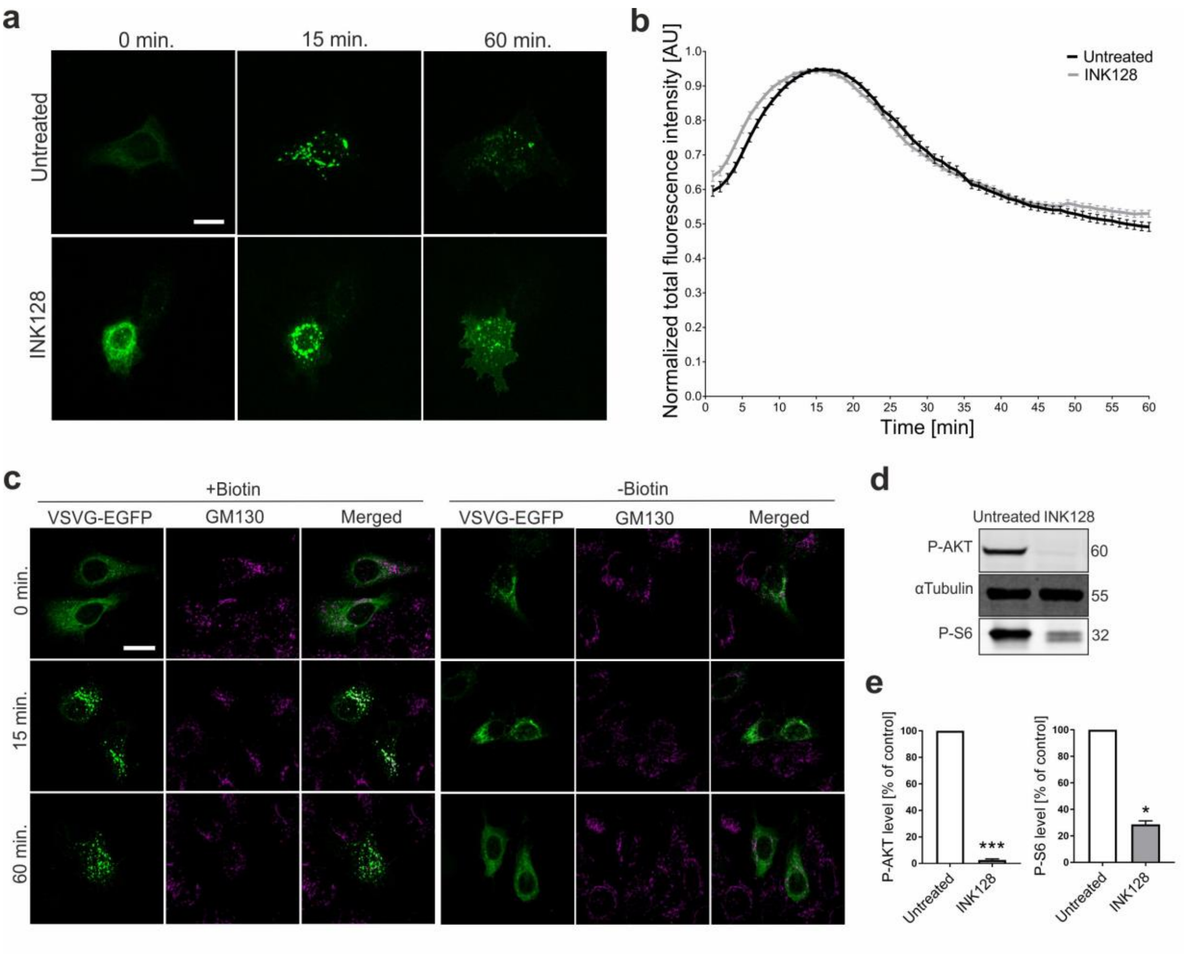

Additional Fig S2. Inhibition of mTOR does not influence secretory trafficking in early-passage MCF7 cells. a. Representative time-lapse confocal images of living MCF7 cells that were transfected with Str-li_VSVGwt-SBP-EGFP and treated with INK128 (300 nM, 30 min) or untreated (control). Secretory trafficking of the fluorescent reporter protein VSVg-EGFP was analyzed using the RUSH system for 60 min after the addition of biotin (time 0). Scale bar = 20 μm. **b.** Quantitative analysis of the live imaging experiments that were performed as in **a**. The plot shows VSVG-EGFP fluorescence intensity in the Golgi apparatus region at each time point, normalized to the maximum value. The data are expressed as the mean for all of the analyzed cells. Error bars indicate SEM. $N = 3$ independent experiments. Number of cells per variant (n): Untreated (45), INK128 (58). **c**. Representative confocal images of MCF7 cells that were transfected with Str-li_VSVGwt-

SBP-EGFP (green) and immunofluorescently stained for the *cis*-Golgi marker GM130 (magenta). Scale bar = 20 μm. **d**. Western blot analysis of phospho-AKT (P-AKT) and phospho-S6 (P-S6) levels in protein lysates from control MCF7 cells or cells after INK128 treatment (300 nM, 30 min). **e**. Quantification of Western blot analysis of P-AKT and P-S6, normalized to tubulin, in protein lysates that were obtained from MCF7 cells that were treated as in **d**. *$p < 0.05$, ***$p < 0.001$ (one-sample *t*-test). Number of independent experiments (*N*): P-AKT (3), P-S6 (2).
